# Deletion of CGI-58 or adipose triglyceride lipase differently affects macrophage function and atherosclerosis[S]

**DOI:** 10.1194/jlr.M052613

**Published:** 2014-12

**Authors:** Madeleine Goeritzer, Stefanie Schlager, Branislav Radovic, Corina T. Madreiter, Silvia Rainer, Gwynneth Thomas, Caleb C. Lord, Jessica Sacks, Amanda L. Brown, Nemanja Vujic, Sascha Obrowsky, Vinay Sachdev, Dagmar Kolb, Prakash G. Chandak, Wolfgang F. Graier, Wolfgang Sattler, J. Mark Brown, Dagmar Kratky

**Affiliations:** * Institute of Molecular Biology and Biochemistry, Center of Molecular Medicine, Medical University of Graz, Graz, Austria; †† Center for Medical Research/Institute of Cell Biology, Histology, and Embryology, Medical University of Graz, Graz, Austria; † Department of Pathology, Section on Lipid Sciences, Wake Forest University School of Medicine, Winston-Salem, NC; § Division of Hypothalamic Research, University of Texas Southwestern Medical Center, Dallas, TX; ** Department of Cellular and Molecular Medicine, Cleveland Clinic Lerner Research Institute, Cleveland, OH

**Keywords:** inflammation, lipid droplets, storage diseases, comparative gene identification-58

## Abstract

Cellular TG stores are efficiently hydrolyzed by adipose TG lipase (ATGL). Its coactivator comparative gene identification-58 (CGI-58) strongly increases ATGL-mediated TG catabolism in cell culture experiments. To investigate the consequences of CGI-58 deficiency in murine macrophages, we generated mice with a targeted deletion of CGI-58 in myeloid cells (macCGI-58^−/−^ mice). CGI-58^−/−^ macrophages accumulate intracellular TG-rich lipid droplets and have decreased phagocytic capacity, comparable to ATGL^−/−^ macrophages. In contrast to ATGL^−/−^ macrophages, however, CGI-58^−/−^ macrophages have intact mitochondria and show no indications of mitochondrial apoptosis and endoplasmic reticulum stress, suggesting that TG accumulation per se lacks a significant role in processes leading to mitochondrial dysfunction. Another notable difference is the fact that CGI-58^−/−^ macrophages adopt an M1-like phenotype in vitro. Finally, we investigated atherosclerosis susceptibility in macCGI-58/ApoE-double KO (DKO) animals. In response to high-fat/high-cholesterol diet feeding, DKO animals showed comparable plaque formation as observed in ApoE^−/−^ mice. In agreement, antisense oligonucleotide-mediated knockdown of CGI-58 in LDL receptor^−/−^ mice did not alter atherosclerosis burden in the aortic root. These results suggest that macrophage function and atherosclerosis susceptibility differ fundamentally in these two animal models with disturbed TG catabolism, showing a more severe phenotype by ATGL deficiency.

Monocyte-derived macrophages are present in virtually all tissues, where they remove apoptotic cells and cellular debris generated by tissue remodelling and/or cellular necrosis. Under pathophysiological conditions, macrophages play a key role during atherogenesis by internalizing modified lipoproteins or lipoprotein remnants that have invaded the vessel wall to form cholesterol-rich foam cells. This (along with other disease-related functions for this immune cell) has prompted profound research into the role of the macrophage, and how its functions are regulated during disease progression (1).

Comparative gene identification-58 (CGI-58) is the coactivator of adipose TG lipase (ATGL), the major TG hydrolase for the initial and rate-limiting step in lipolysis (2). Lipolysis has been extensively studied in adipocytes, where under basal conditions CGI-58 binds to the surface of lipid droplets through interaction with perilipin1. Hormonal stimulation of lipolysis leads to phosphorylation of perilipin1 and hormone-sensitive lipase, resulting in the release of CGI-58 from perilipin1 to interact with and activate ATGL, which then converts TG to diacylglycerol and FA (3). Both mice and humans affected with ATGL or CGI-58 deficiency suffer from systemic TG accumulation, a condition called neutral lipid storage disease (NLSD) in humans (4). Of note, specific phenotypical alterations are observed depending on whether ATGL or CGI-58 is defective. The most apparent difference is the severe epidermal skin defect observed in mice and humans with CGI-58 deficiency (2, 5), which is absent in both species lacking ATGL (6, 7). This finding resulted in different classifications of the respective human diseases, namely NLSD with myopathy in ATGL deficiency (6), whereas CGI-58 deficiency leads to NLSD with ichthyosis (8). Several ongoing studies using tissue-specific CGI-58 and ATGL-deficient (−/−) mice aim at studying the proteins’ shared and individual contribution to lipid metabolism. In addition, functional differences observed in hepatocytes between mice with CGI-58 and ATGL deficiency argue for an ATGL-independent function of CGI-58 in this cell population (9, 10) and maybe other tissues as well (11).

Because CGI-58^−/−^ mice die shortly after birth due to a severe skin barrier defect (5), we generated myeloid-specific CGI-58 (macCGI-58)^−/−^ mice to investigate the consequences of CGI-58 deficiency in macrophages. In the present study, we examined: *i*) whether CGI-58^−/−^ macrophages mimic the TG accumulation phenotype observed in ATGL^−/−^ macrophages; *ii*) whether CGI-58 deficiency affects macrophage function; and *iii*) whether the altered phenotype culminates in increased atherosclerosis susceptibility in macCGI-58/ApoE-double KO (DKO) animals. We have previously shown that loss of ATGL in macrophages affects macrophage phenotype and function, such as TG-rich lipid droplet accumulation, increased apoptosis (12) and endoplasmic reticulum (ER) stress (13), reduced migration (14), and decreased phagocytosis ability (15). In addition, transplantation of ATGL^−/−^ bone marrow into LDL receptor (LDLR)^−/−^ mice revealed that the lack of ATGL in immune cells attenuates atherosclerosis susceptibility (16). Being the coactivator of ATGL, we predicted that the absence of CGI-58 in macrophages leads to TG-rich lipid droplet accumulation. We hypothesized that loss of the ATGL coactivator CGI-58 in myeloid cells affects macrophage function in vitro and in vivo and impacts atherosclerosis susceptibility.

## MATERIALS AND METHODS

### Animals and diets

Mice with a targeted deletion of CGI-58 in myeloid cells (macCGI-58^−/−^ mice) were generated by crossing CGI-58^flox/flox^ mice (17) (provided by Dr. Guenther Haemmerle, University of Graz, Austria) with transgenic mice that express Cre recombinase under the control of the murine M lysozyme promoter (18) (LysMCre, C57BL/6 background; provided by Dr. Thomas Ruelicke, University of Veterinary Medicine, Vienna, Austria). CGI-58^flox/flox^ [wild-type (Wt)] mice were used as controls. Experiments were performed with female animals. To investigate atherosclerosis susceptibility, we generated CGI-58^flox/flox^/ApoE^−/−^ (designated as ApoE^−/−^) and macCGI-58/ApoE-DKO mice by crossing CGI-58^flox/flox^ and macCGI-58-KO mice with ApoE^−/−^ mice (Jackson Laboratory, Bar Harbor, ME). For genotyping, the following primers were used: CGI-58^flox/flox^-forward, 5′-GTCATGGTTGT­GG­GGAAATC-3′; CGI-58^flox/flox^-reverse, 5′-GACTGGAAG­GA­TTT­GA­GGGG-3′; Cre-mut, 5′-CCCAGAAATGCCAGATTACG-3′; Cre-comm, 5′-CTTGGGCTGCCAGAATTTCTC-3′; Cre-Wt, 5′-TTACAGTCGGCCAGGCTGAC-3′; ApoE-forward, 5′-GCCTAGCCGAG­GGA­GA­GCCG-3′; ApoE-reverse, 5′-TGTGACTTGGGAGCTCTGCAGC-3′; and ApoE-neo, 5′-GCCGCCCCGACTGCATCT-3′.

Female mice were either fed a standard chow diet [containing 4% fat and 21% protein (R/M H; Ssniff, Soest, Germany)], challenged with a Western type diet (WTD) [TD88137mod; 21% fat, 0.2% cholesterol (Ssniff)] or a high-fat/high-cholesterol diet (HF/HCD) (E15126-34 EF R/M; 30% fat, 1% cholesterol) for 10–30 weeks starting at the age of 4–6 weeks. Mice were kept with water ad libitum on a regular light-dark cycle (12 h light, 12 h dark) in a clean environment. Body weights were measured weekly and plasma lipid parameters once a month.

For atherosclerosis studies using antisense oligonucleotide (ASO)-mediated knockdown of CGI-58, 6-week-old male LDLR^−/−^ mice were fed a diet enriched in 0.2% (w/w) cholesterol and 20% of energy as lard for 16 weeks in conjunction with weekly injections (50 mg/kg) of either a nontargeting control ASO or CGI-58 ASO, as previously described (9). Plasma samples were collected by submandibular vein puncture at baseline (chow-fed animals, 6 weeks of age), and after 4, 8, and 16 weeks of diet and ASO treatment for subsequent lipid and lipoprotein analyses. The majority of animal experiments were performed according to the standards set by the Austrian Federal Ministry of Science and Research, Division of Genetic Engineering and Animal Experiments, Vienna, Austria (BMWF-66.010/0039-II/10b/2009, BMWF-66.010/0057-II/3b/2011). The ASO-mediated knockdown studies of CGI-58 were conducted in an American Association for Accreditation of Laboratory Animal Care-approved animal facility, and all experimental protocols were approved by the Institutional Animal Care and Use Committee at either the Wake Forest University School of Medicine or the Cleveland Clinic Lerner Research Institute.

### Cell culture

Peritoneal macrophages were collected after an ip injection of 2.5 ml 3% thioglycolate. After 3 days, the peritoneum was flushed with 10 ml PBS containing 1 mM EDTA. The cells were cultivated in DMEM (Gibco, Invitrogen, Carlsbad, CA) containing 10% lipoprotein-deficient serum (LPDS) and 1% penicillin/streptomycin for 2–3 h. Thereafter, the cells were washed three times with prewarmed PBS and the adherent cells (macrophages) were cultured in DMEM containing 25 mM glucose, 4 mM glutamine, 1 mM pyruvate, 10% LPDS, and 1% penicillin/streptomycin for 24 h. Bone marrow-derived macrophages were isolated from femur and tibia flushed with sterile PBS. Cells were cultured in DMEM containing 10% LPDS, 1% penicillin/streptomycin, and 10 ng/ml macrophage colony-stimulating factor for 7 days.

To assess in vitro lipopolysaccharide (LPS)-induced acute-phase response, macrophages were treated with saline (control) or LPS (100 ng/ml) for 16 h. IL-6 concentrations in supernatants were determined by ELISA (Enzo Life Sciences, Lausen, Switzerland).

For the studies using ASO-mediated knockdown, elicited peritoneal macrophages were collected 4 days after injection of 1 ml of 10% thioglycolate into the peritoneal cavities of C57BL/6 mice that had been treated with ASOs and fed a chow diet for 6 weeks, as previously described (19). Following 2 h of culture, nonadherent cells were removed by washing three times with PBS, and remaining adherent macrophages were harvested for Western blotting using methods previously described (9).

### Plasma lipid parameters

Blood was collected from 12 h-fasted mice or from 12 h-fasted/2 h-refed mice, and plasma was prepared by centrifugation at 5,200 *g* for 7 min at 4°C. Plasma TG, total cholesterol (TC), free cholesterol (FC), and nonesterified FA concentrations were measured enzymatically by commercially available kits (DiaSys, Holzheim, Germany; Wako Chemicals GmbH, Neuss, Germany). For atherosclerosis studies using ASO-mediated knockdown of CGI-58, total plasma concentrations of TC and TG were measured enzymatically by commercially available kits (Wako Chemicals, Richmond, VA). In addition, plasma lipoproteins were separated by fast protein liquid chromatography, and cholesterol concentrations in lipoprotein fractions were measured using an enzymatic assay as previously described (19).

### Lipid parameters in macrophages

Macrophages were plated for 2 h in serum-free DMEM. After washing the cells three times with PBS, lipids were extracted with 2 ml hexane:isopropanol (3:2, v:v) for 1 h at 4°C. One hundred microliters of 1% Triton X-100 in chloroform were added and the lipid extract was dried under a stream of nitrogen. The samples were dissolved in 100 μl ddH_2_O for 15 min at 37°C in a water bath. TG, TC, and FC concentrations were measured enzymatically by using 30 μl of the sample with the above mentioned kits. The readings were normalized to protein concentrations. Protein was quantitated using a Lowry assay (Bio-Rad Laboratories, Hercules, CA) after dissolving the proteins of cells in 2 ml NaOH (0.3 M) for 2 h at room temperature.

FA composition in the TG fraction was quantitated by GC-flame ionization detection. Briefly, lipid extracts were separated by thin layer chromatography (hexane:diethylether:acetic acid, 70:30:1, v:v:v) and the band comigrating with tri-C16:0 TG was scraped, extracted with CHCl_3_/methanol (2:1, v:v), dried, and transesterified in BF_3_/toluene. Pentadecanoic acid was used as internal standard. Separation and quantitation were performed as previously described (20).

### Nile Red staining and fluorescence microscopy

Macrophages were plated on chamber slides in DMEM containing 10% LPDS and 1% penicillin/streptomycin for 24 h. Cells were washed three times with PBS and fixed with 10% formalin (30 min). Lipid droplets were visualized after Nile Red staining (2.5 μg/ml) by confocal laser scanning microscopy using an LSM 510 META microscope system (Carl Zeiss GmbH, Vienna, Austria). Pictures (×63 magnification) were taken at excitation 543 nm and signals were recorded using a 560 nm long pass filter.

### TG and CE hydrolase activity assays

Macrophages were lysed with 100 μl of lysis buffer [100 mM potassium phosphate, 250 mM sucrose, 1 mM EDTA, 0.1 mM DTT (pH 7)], sonicated on ice twice for 10 s with 10 s interval, and protein concentrations were measured using a Lowry assay (BioRad Laboratories). The TG substrate contained 17 nmol triolein/assay and 2,000 cpm/nmol of [9,10-^3^H(N)]triolein (Perkin Elmer, Waltham, MA). The cholesteryl ester (CE) substrate contained 20 nmol cholesteryl oleate per assay and 1,000 cpm/nmol of cholesteryl [1-^14^C]oleate (Amersham Biosciences, Piscataway, NJ). Fifty micrograms of protein from cell lysates was mixed with 100 μl of substrate and incubated in a water bath for 1 h at 37°C. The reaction was stopped by the addition of 3.25 ml stop solution (methanol:chloroform:n-heptane, 10:9:7, v:v:v) and 1 ml of 0.1 M potassium carbonate and 0.1 M boric acid (pH 10.5) (21). The tubes were vortexed for 10–15 s and centrifuged at 800 *g* for 20 min at 4°C. The radioactivity in 1 ml of the upper phase was determined by liquid scintillation counting, and the release of FAs was calculated.

### LPL activity in macrophages

Macrophages were incubated in 6-well plates with 300 μl medium, 2% FA-free BSA (Sigma-Aldrich, St. Louis, MO), and 2 units/ml of heparin for 1 h at 37°C under continuous shaking. For the substrate preparation per sample, 0.6 μCi [^3^H]triolein, 920 ng glycerol trioleate, and 0.1% Triton X-100 in chloroform were evaporated under a stream of nitrogen. Forty microliters of 1 M Tris-HCl (pH 8.6) and 80 μl ddH_2_O were added, and the mixture was sonicated six times (1 min on and 1 min off) on ice. Then 40 μl of heat-inactivated human serum containing ApoC-II as activator (obtained from a pool of donors, heated at 50°C for 1 h, and stored at 20°C) and 40 μl of 10% FA-free BSA were added to the substrate. Analysis was performed as previously described (15).

### Real time PCR

Total RNA from macrophages was isolated using a PerfectPure RNA cultured cell kit (5Prime, Hamburg, Germany). RNA concentrations were measured at 260 nm on a NanoDrop instrument (Thermo Scientific, Wilmington, DE). Two micrograms of total RNA were reverse transcribed by using the high capacity cDNA reverse transcription kit (Applied Biosystems, Foster City, CA). Quantitative real time PCR was performed on a LightCycler 480 (Roche Diagnostics, Rotkreuz, Switzerland) using the QuantifastTM SYBR^®^ Green PCR kit (Qiagen, Hilden, Germany). Amplification of murine hypoxanthine-guanine phosphoribosyltransferase (HPRT) as housekeeping gene was performed on all samples as internal controls for variations in mRNA amounts. Expression profiles and associated statistical parameters were determined using the public domain program Relative Expression Software Tool-REST 2008 (22). Primer sequences are listed in the supplementary material.

### Western blotting

Protein samples of lysed macrophages from the different genotypes (40 or 50 μg protein/lane) were separated by SDS-PAGE (15%). Proteins were transferred to polyvinylidene difluoride or nitrocellulose membranes. Blots were incubated with monoclonal anti-mouse antibodies against β-actin (1:20,000) (Santa Cruz, Heidelberg, Germany), ABHD5/CGI-58 (1:1,000) (Abnova GmbH, Heidelberg, Germany), and CCAAT/enhancer-binding protein homologous protein (CHOP) (1:1,000) (Cell Signaling Technology, Danvers, MA), or anti-rabbit polyclonal antibodies against Bax (1:1,000), cytochrome C (1:1,000), inositol-requiring enzyme 1α (IRE1α) (1:1,000), CHOP (1:1,000), and ATGL (1:200) (Cell Signaling Technology). HRP-conjugated goat anti-rabbit (1:5,000) or rabbit anti-mouse antibodies (1:1,000) (Dako, Glostrup, Denmark) were visualized by enhanced chemiluminescence detection (Clarity^TM^ Western ECL substrate; Bio-Rad) using a ChemiDoc^TM^ MP imaging system (Bio-Rad).

### Mitochondrial respiration measurement

Macrophages were plated in XF96 polystyrene cell culture microplates (Seahorse Bioscience^®^, North Billerica, MA) at a density of 60,000 cells per well. After 24 h, cells were washed and preincubated for 30 min in XF assay medium supplemented with sodium pyruvate (1 mM) with or without glutamine (2 mM) and glucose (25 mM) at 37°C in a nonCO_2_ environment. The oxygen consumption rate (OCR) was subsequently measured every 7 min using an XF96 extracellular flux analyzer (Seahorse Bioscience^®^). A standard protocol with 15 min basal measurement followed by 10 μM oligomycin, addition of 0.3 μM carbonyl cyanide *p*-trifluoromethoxyphenylhydrazone (FCCP), and 2.5 μM antimycin A was performed. Oxygen consumption was either normalized to protein content (pmol O_2_/min × μg protein) or expressed as a percentage of the maximal mitochondrial respiration in the presence of 0.3 μM FCCP.

### Phagocytosis of fluorescein-labeled *Escherichia coli* particles

Macrophages were plated in black 96-well μClear plates (Greiner Bio-One GmbH, Solingen, Germany). After 24 h of preincubation in DMEM, 10% LPDS, and 25 mM glucose, cells were incubated in DMEM, 10% LPDS, and 0, 6, or 25 mM glucose for 1 h, respectively. Cells were washed and incubated with 100 μl of fluorescein-labeled *E. coli* BioParticles (Vybrant^TM^ phagocytosis assay, Molecular Probes, Invitrogen; suspended in Hanks’ balanced salt solution; 2 h). The suspension was removed and subsequently 100 μl of trypan blue was added (1 min) to quench the extracellular probe. After aspiration of trypan blue, the fluorescence was measured at 484 nm (excitation) and 535 nm (emission) on a Victor 1420 multilabel counter (PerkinElmer Life Sciences, Turku, Finland). Fluorescence was normalized to the protein content of each well.

To analyze phagocytosis in vivo, mice were injected intraperitoneally with 200 μl of fluorescein-labeled *E. coli* BioParticles suspended in Hanks’ balanced salt solution. After 2 h, macrophages were collected by flushing the peritoneal cavity with 10 ml PBS containing 1 mM EDTA and incubated in DMEM containing 25 mM glucose and 10% LPDS for 90 min. The cells were washed three times with PBS, and fluorescence was measured before and after adding trypan blue to obtain total and intracellular fluorescence, respectively. Experimental readings were normalized to protein content.

### Apoptosis assay

Apoptosis was assayed by annexin V and propidium iodide (PI) costaining (Annexin-V-Fluor staining kit; Roche, Vienna, Austria). Two hundred thousand cells were washed twice with 200 μl PBS; 50 μl staining buffer was added and cells were incubated for 10 min. Macrophages were immediately analyzed on a FACScalibur flow cytometer (BD Biosciences, San José, CA).

### Glucose tolerance test

Animals were fasted for 6 h (6 AM to 12 PM) with free access to drinking water. Blood was taken from the tail vein before and 15, 30, 60, 120, and 180 min after an ip injection of glucose (2.0 g/kg body weight). Glucose concentrations from blood were determined using a portable glucometer (AccuCheck).

### Preparation of histological sections and lesion analysis

We analyzed atherosclerotic lesions in the aortic root and aorta of ApoE^−^*^/^*^−^ and macCGI-58/ApoE-DKO animals after 10 weeks of HF/HCD feeding. Mice were euthanized and the arterial tree was perfused in situ with PBS (100 mm Hg) for 10 min via a cannula in the left ventricular apex. Mice were perfused with 10% formalin (Carl Roth GmbH, Vienna, Austria) for 15 min. After fixing the hearts in 10% formalin, serial sections (8 μm) were cut (HM 560 Cryo-Star; Microm International GmbH, Walldorf, Germany). Images of the atherosclerotic lesion areas in Oil Red O-stained (Sigma-Aldrich) sections were taken with ScanScope T3 whole slide scanner (Aperio Technologies, Bristol, UK). Plaque areas were quantitated by ImageJ software. Mean lesion area was calculated from 10 consecutive Oil Red O-stained sections, starting at the appearance of the tricuspid valves. Sections were stained immunohistochemically for the presence of macrophages using a monoclonal rat anti-mouse Moma-2 antibody (1:600) (Acris, Hiddenhausen, Germany), as well as for collagen content using Masson’s trichrome staining kit (Sigma-Aldrich). For en face analysis in macCGI-58/ApoE-DKO mice, aortas were dissected and plaques were stained with Oil Red O as described recently (23). Images were analyzed using ImageJ software.

For studies in ASO-treated mice, hearts and aortae were carefully separated and hearts were immediately slow frozen in OCT for cross-sectional lesion analysis of the aortic sinus. Whole aortae were fixed in 10% neutral buffered formalin for subsequent en face analysis. For atherosclerosis quantification in the aortic sinus, histological cross-sections were stained with Oil Red O. Images were captured using a Leica DMR microscope (W. Nuhsbaum Inc., McHenry, IL) equipped with a Q imaging Retiga EX camera. Images were analyzed using Image-Pro Plus 7.0 (MediaCybernetics, Rockville, MD). Additionally, en face lesion area was determined for the whole aorta as described previously (19).

### Cellular cholesterol efflux

Macrophages were incubated with 50 μg acLDL [preloaded with 0.5 μCi/ml [^3^H]cholesterol (ARC Inc., St. Louis, MO)] and 30 μg/ml nonlabeled cholesterol in DMEM/0.2% FA-free BSA for 32 h at 37°C. After washing the cells twice with PBS, the cells were cultivated for 16 h in equilibration medium (DMEM/0.2% FA-free BSA). We determined cholesterol efflux after incubating the cells in DMEM/0.2% FA-free BSA in the absence or presence of 15 μg/ml ApoA-I (Calbiochem, La Jolla, CA) or 100 μg/ml HDL_3_. Radioactivity in 80 μl medium and in the cells was measured by scintillation counting after 1, 3, 6, and 9 h of incubation. Cholesterol efflux is expressed as the percentage of total cell [^3^H]cholesterol present in the medium after 1, 3, 6, and 9 h. Basal efflux in the absence of ApoA-I and HDL_3_ was subtracted from the data shown.

### Electron microscopy

Macrophages were cultured on an Aclar film and fixed in 2.5% (w/v) glutaraldehyde and 2% (w/v) formaldehyde in 0.1 M phosphate buffer (pH 7.4, 2 h), postfixed in 2% (w/v) osmium tetroxide (2 h) at room temperature, dehydrated in graded series of ethanol, and embedded in a TAAB epoxy resin.

Ultrathin sections (75 nm) were cut with a Leica UC 7 Ultramicrotome and stained with lead citrate (5 min) and with uranyl acetate (15 min). Images were taken using a FEI Tecnai G2 20 transmission electron microscope (FEI, Eindhoven, The Netherlands) with a Gatan ultrascan 1000 CCD camera. Acceleration voltage was 120 kV.

### Statistics

Statistical analyses were performed using GraphPad Prism 5.0 software. Significance was calculated by Student’s unpaired *t*-test or ANOVA, followed by Bonferroni correction. Data are presented as mean values ± SEM. Significance levels were set at *P* < 0.05 (*), *P* ≤ 0.01 (**), and *P* ≤ 0.001 (***).

## RESULTS

### Plasma lipid parameters, body weight, and glucose levels are unaffected in macCGI-58^−/−^ mice

**Fig. 1.**
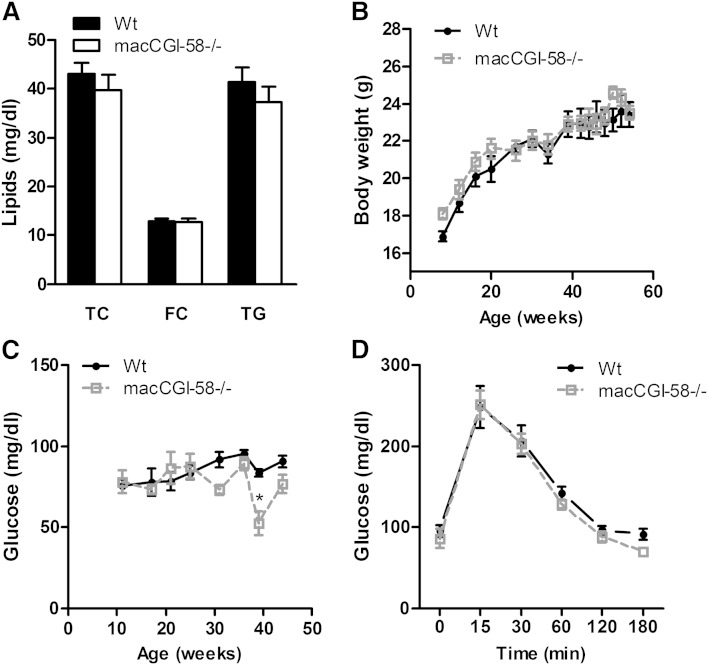
Unchanged plasma lipid parameters, body weight, glucose concentrations, and glucose tolerance in macCGI-58^−/−^ mice. Plasma parameters (A) and body weight (B) of overnight fasted 12–14-week-old female Wt and macCGI-58^−/−^ mice fed a standard chow diet. C: Glucose concentrations in female mice aged 11–46 weeks of age. D: Glucose tolerance test of 34–35-week-old female Wt and macCGI-58^−/−^ mice fed WTD. Data are shown as mean values ± SEM (n = 4–5).

macCGI-58^−/−^ mice are viable with no apparent changes in skin phenotype (not shown). We observed no differences in lipid parameters and body weight between female Wt and macCGI-58^−/−^ mice, neither on chow (**Fig. 1A, B**) nor on WTD (data not shown). Glucose concentrations were decreased at 39 weeks of age but comparable to control levels at all other time points (Fig. 1C). Glucose tolerance in mice fed WTD was unchanged as well (Fig. 1D). We also observed no significant differences between both genotypes when we repeated the experiments in male mice (data not shown).

### Decreased TG hydrolase activity and TG-rich lipid droplet accumulation in CGI-58^−/−^ macrophages

**Fig. 2.**
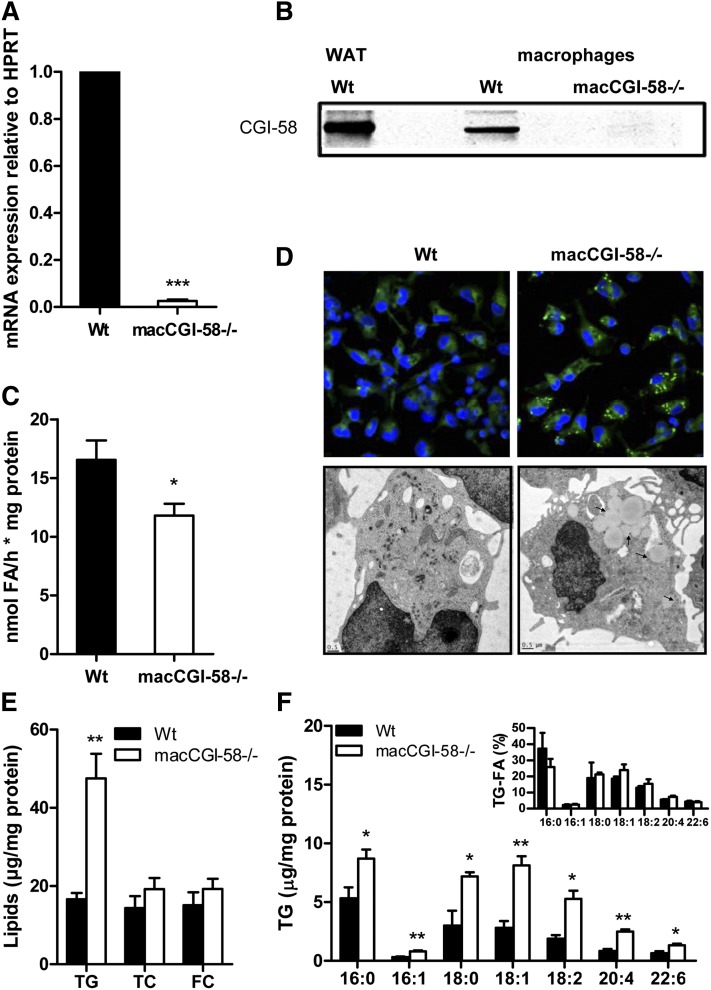
Decreased TG hydrolase activity and TG-rich lipid droplet accumulation in CGI-58^−/−^ macrophages. A: mRNA expression of CGI-58 in Wt and CGI-58^−/−^ macrophages determined by real time PCR, including normalization to HPRT, expressed as mean values + SEM, performed in duplicate (n = 3–4). B: Western blot analysis of CGI-58 protein expression in Wt white adipose tissue (WAT) as well as Wt and CGI-58^−/−^ macrophages (50 μg protein per lane). C: TG hydrolase activities in cell lysates of Wt and CGI-58^−/−^ macrophages are presented as mean values + SEM, performed in duplicate (n = 4). D: Representative fluorescent microscopy images after Nile Red staining and electron micrographs of Wt and CGI-58^−/−^ macrophages. Lipid droplets in the CGI-58^−/−^ macrophages are indicated by arrows. E: TG, TC, and unesterified FC concentrations in lipid extracts of macrophages presented as mean values + SEM (n = 4–5). F: FA composition in TG of macrophages after separation by thin layer chromatography determined by GC-flame ionization detection. Data are presented as mean values + SEM (n = 4). Inset: TG-FA distribution in percentage. *P < 0.05; **P ≤ 0.01; ***P ≤ 0.001.

We confirmed the absence of CGI-58 expression in macrophages by real time PCR (**Fig. 2A**) and Western blotting (Fig. 2B). TG hydrolase activity of lysates was significantly (−29%) decreased in CGI-58^−/−^ compared with Wt macrophages (Fig. 2C). CGI-58^−/−^ macrophages showed an increased number of lipid droplets as evidenced by immunofluorescence and electron microscopy (Fig. 2D). Biochemical measurements revealed a specific accumulation of TG (Fig. 2E). CE hydrolase activities were comparable in macrophages from CGI-58^−/−^ and Wt mice (supplementary Fig. I), which is in accordance with unaltered TC and unesterified FC concentrations (Fig. 2E). Analysis of FA composition within TG revealed increased concentrations of all FA species analyzed (Fig. 2F). Unchanged relative distribution of FAs within TG indicates that hydrolysis preferences are comparable between macrophages of both genotypes (inset Fig. 2F).

To investigate whether CGI-58 deficiency in macrophages results in a compensatory upregulation of genes involved in intra- and extracellular TG hydrolysis, we determined mRNA expression of ATGL, hormone-sensitive lipase, lysosomal acid lipase, and LPL. We found no significant differences in the mRNA expression of these genes in CGI-58^−/−^ compared with Wt macrophages (supplementary Fig. IIA). ATGL protein expression was unchanged as well (supplementary Fig. IIB). To further address whether additional metabolic changes interfere with TG homeostasis in CGI-58^−/−^ macrophages, we examined LPL activity. Like in ATGL^−/−^ macrophages, LPL activity was unchanged in CGI-58^−/−^ macrophages (supplementary Fig. IIC).

### Apoptosis and ER stress are not induced in CGI-58^−/−^ macrophages

**Fig. 3.**
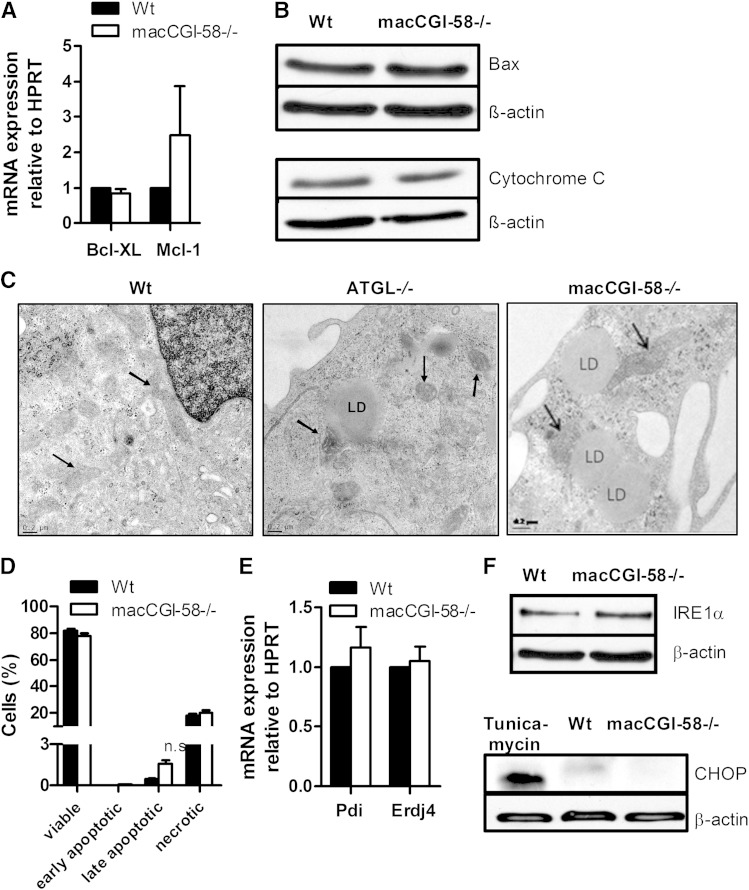
Apoptosis, mitochondrial fragmentation, and ER stress are not induced in CGI-58^−/−^ macrophages. A: mRNA expression of the anti-apoptotic genes Bcl-XL and Mcl-1 in Wt and CGI-58^−/−^ macrophages determined by real time PCR, including normalization to HPRT. Expression in Wt macrophages was arbitrarily set to one. Data are expressed as mean values + SEM, performed in duplicate (n = 5). B: Western blot analysis of Bax protein expression in macrophages (40 μg protein per lane). The cytosolic fraction was blotted for cytochrome C expression. C: Representative electron micrographs of mitochondria (indicated by arrows) in Wt, ATGL^−/−^, and CGI-58^−/−^ macrophages. D: Quantifications of the total amount of annexin V-positive (early apoptotic), annexin V/PI-positive (late apoptotic), and PI-positive (necrotic) cells shown as mean values + SEM, performed in duplicate (n = 5). Data represent the percentage of cells stained with annexin V and/or PI. E: mRNA expression of the ER-resident chaperones Pdi and Erdj4. Data are expressed as mean values + SEM, performed in duplicate (n = 5). IRE1α (F) and CHOP (G) protein expression determined by Western blotting analyses. As positive control for CHOP expression Wt macrophages were treated with the ER stress inducer, tunicamycin. LD, lipid droplet; n.s., not significant.

We have previously shown that the mitochondrial apo­ptosis pathway is induced in ATGL^−/−^ macrophages (12). mRNA expression levels of the two anti-apoptotic markers Bcl-XL and Mcl-1, however, were unchanged in CGI-58^−/−^ macrophages (**Fig. 3A**). Furthermore, Western blotting analysis revealed no differences in the protein expression of Bax and cytosolic cytochrome C (Fig. 3B), which are key players in mitochondria-dependent apoptosis (24). Unlike ATGL^−/−^ macrophages, mitochondria in CGI-58^−/−^ macrophages are electron dense and have intact cristae, as in Wt macrophages (Fig. 3C). Finally, Annexin V/PI staining revealed the same amount of alive, early apoptotic, and necrotic cells. The number of late apoptotic cells was increased by 3.6-fold in CGI-58^−/−^ macrophages but lacked statistical significance (Fig. 3D).

Next, we investigated whether ER stress might be activated in CGI-58^−/−^ macrophages due to the TG-rich lipid droplet accumulation, as observed in ATGL^−/−^ macrophages (13). These analyses revealed unchanged mRNA levels of the ER-resident chaperones Pdi and Erdj4 (Fig. 3E), unaltered protein expression of IRE1α (Fig. 3F), which is responsible for the X-box-binding protein 1 splicing during ER stress (13), and no protein expression of the cell death executor CHOP (Fig. 3G) in CGI-58^−/−^ macrophages (25). These findings demonstrate that CGI-58^−/−^ macrophages lack any signs of mitochondrial apo­ptosis, ER stress, and mitochondrial dysfunction as observed in ATGL^−/−^ macrophages.

### Decreased phagocytosis and mitochondrial respiration in CGI-58^−/−^ macrophages

**Fig. 4.**
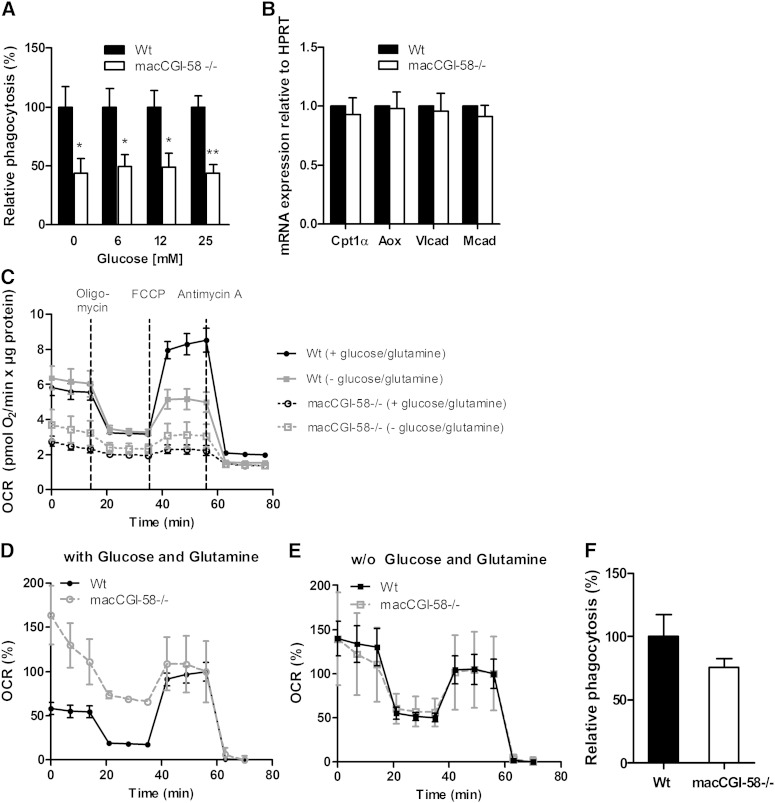
Reduced phagocytosis capacity and mitochondrial respiration of CGI-58^−/−^ macrophages. A: Macrophages from Wt and macCGI-58^−/−^ mice were cultivated in DMEM/10% LPDS containing 0, 6, and 25 mM glucose for 1 h. Phagocytosis of fluorescein-labeled *E. coli* particles is presented as mean values + SEM of two independent experiments performed in triplicate (n = 8–9). Phagocytosis of Wt cells was arbitrarily set to 100%. *P < 0.05; **P ≤ 0.01. B: mRNA expression of the PPARα target genes Ctp1α, Aox, Vlcad, and Mcad, including normalization to HPRT, was determined by real time PCR. Data are expressed as means + SEM, performed in duplicate (n = 5). C: The OCR of Wt (continuous lines) and CGI-58^−/−^ macrophages (dotted lines) in the presence (black) or absence (gray) of 25 mM glucose and 2 mM L-glutamine normalized to protein content. As indicated, cells were treated with 10 μM oligomycin, 0.3 μM FCCP, and 2.5 μM antimycin A. Data are presented as mean values ± SEM of triplicate repeats (n = 3–4). D, E: OCR calculated as percentage of maximal mitochondrial respiration of Wt and CGI-58^−/−^ macrophages in the presence (D) or absence (E) of 25 mM glucose and 2 mM L-glutamine. Data are presented as mean values ± SEM of triplicate repeats (n = 3–4). F: Fluorescein-labeled *E. coli* particles (200 μl) were injected into Wt and macCGI-58^−/−^ mice. After 2 h, macrophages were isolated and assayed for internalized fluorescence after quenching of extracellular fluorescence by trypan blue. Phagocytosis of Wt macrophages was arbitrarily set to 100%. Relative phagocytosis is presented as mean values + SEM (n = 5).

To address whether the absence of CGI-58 in macrophages affects phagocytosis comparable to ATGL deficiency (15), we performed in vivo and in vitro phagocytosis assays. We observed significantly decreased phagocytosis capacity in CGI-58^−/−^ compared with Wt macrophages, independent of glucose availability (**Fig. 4A**). To elucidate whether decreased β-oxidation might contribute to the reduced phagocytic capacity of CGI-58^−/−^ macrophages, we analyzed the expression of PPARα target genes. mRNA expression levels of carnitine palmitoyl-transferase 1α (Cpt1α), fatty acyl-CoA oxidase (Aox), very long chain acyl-CoA dehydrogenase (Vlcad), and medium chain acyl-CoA dehydrogenase (Mcad), however, were comparable between CGI-58^−/−^ and Wt macrophages (Fig. 4B). Next, we analyzed whether basal and maximal respiration rates are changed in mitochondria of CGI-58^−/−^ macrophages in the presence or absence of glucose and glutamine. Measurement of the absolute OCR revealed that mitochondria of CGI-58^−/−^ macrophages respire less compared with mitochondria from Wt mice in both conditions (Fig. 4C). Relative OCR (presented as percent of maximal respiration) demonstrates that mitochondria from CGI-58^−/−^ macrophages are still responsive to oligomycin treatment and chemical uncoupling by FCCP (Fig. 4D, E). In addition, we observed unchanged mitochondrial surface area (supplementary Fig. IIIA) and protein expression of cyclooxygenase 4, complex Va, and complex III (supplementary Fig. IIIB) in CGI-58^−/−^ macrophages. In vivo, phagocytosis ability tended to be decreased in macCGI-58^−/−^ mice (25%) (Fig. 4F). This effect, however, reached no statistical significance.

### CGI-58^−/−^ macrophages polarize toward M1

**Fig. 5.**
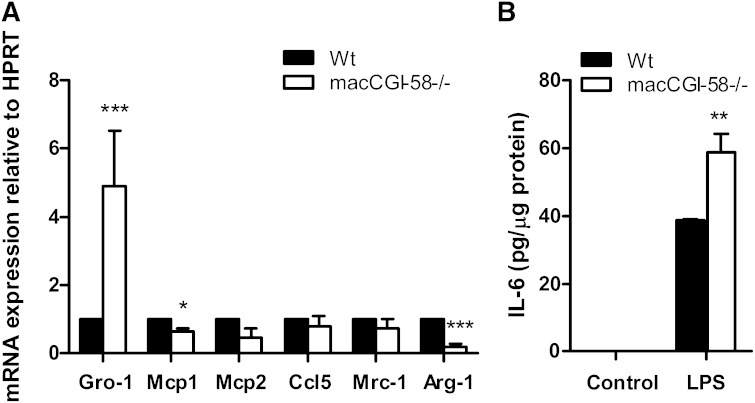
CGI-58^−/−^ macrophages polarize toward a pro-inflammatory M1-like phenotype. A: mRNA expression of Gro-1, Mcp1, Mcp2, Ccl5, Mrc-1, and Arg-1 in Wt and CGI-58^−/−^ macrophages determined by real time PCR, including normalization to HPRT. Data are presented as mean values + SEM, performed in duplicate (n = 5). *P < 0.05; ***P ≤ 0.001. B: Macrophages were treated with saline (control) or LPS (100 ng/ml) for 16 h. IL-6 secretion in the supernatant was determined by ELISA. Data represent mean values + SEM (n = 3–5). **P ≤ 0.01.

Macrophages are a heterogeneous and phenotypically polarized cell population consisting of classically activated (pro-inflammatory) M1 macrophages and alternatively activated M2 macrophages with anti-inflammatory properties (26). We have previously shown that ATGL^−/−^ macrophages adopt an anti-inflammatory M2-like phenotype (14). To investigate the polarization phenotype of CGI-58^−/−^ macrophages, we determined mRNA levels of pro- and anti-inflammatory cytokines, respectively. These analyses revealed an upregulation of the pro-inflammatory cytokine Gro-1 (4.9-fold), downregulation of the pro-inflammatory cytokine Mcp1 (by 36%), and unchanged mRNA expression of Mcp2, Ccl5, and Mrc-1 in CGI-58^−/−^ compared with Wt macrophages (**Fig. 5A**). In line with this, mRNA expression of the M2 marker Arg-1 (27) was down­regulated (by 81%). Furthermore, increased IL-6 concentrations in the supernatant of LPS-treated CGI-58^−/−^ compared with Wt cells (Fig. 5B) support the pro-inflammatory M1-like polarized pattern of CGI-58^−/−^ macrophages.

### Unchanged atherosclerotic lesion formation in macCGI-58/ApoE-DKO mice

**Fig. 6.**
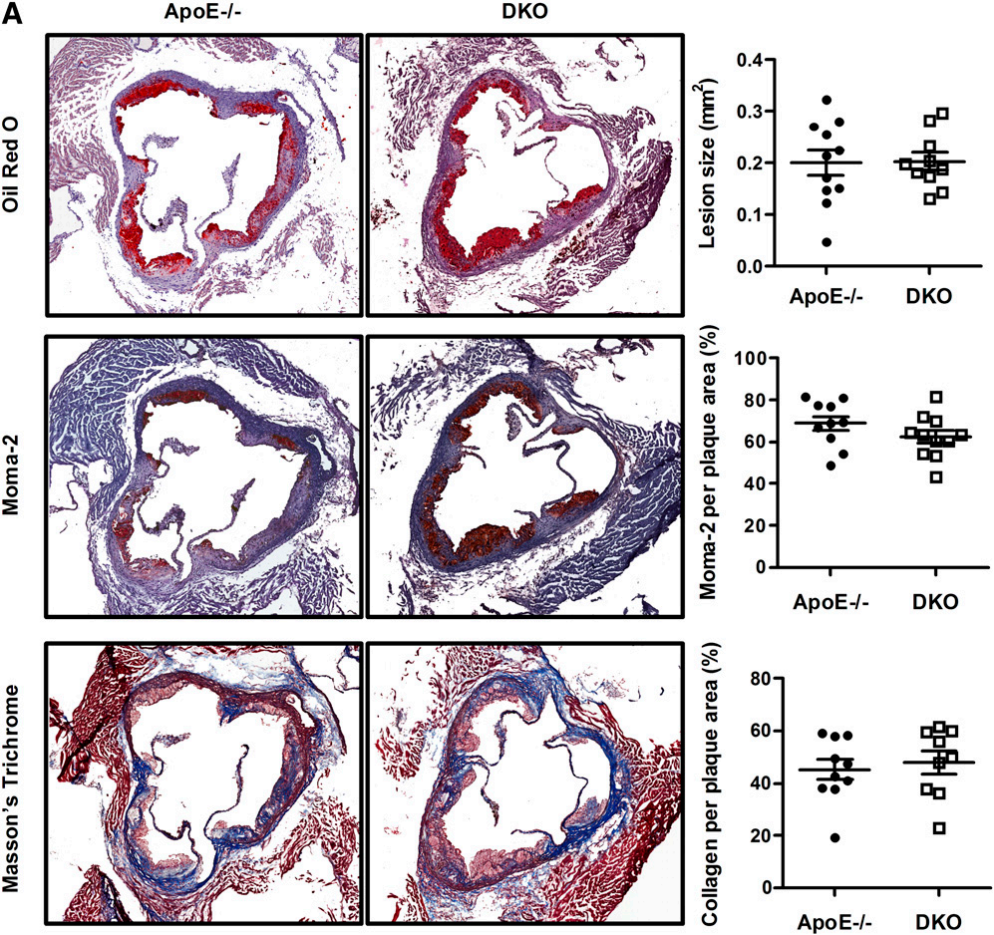

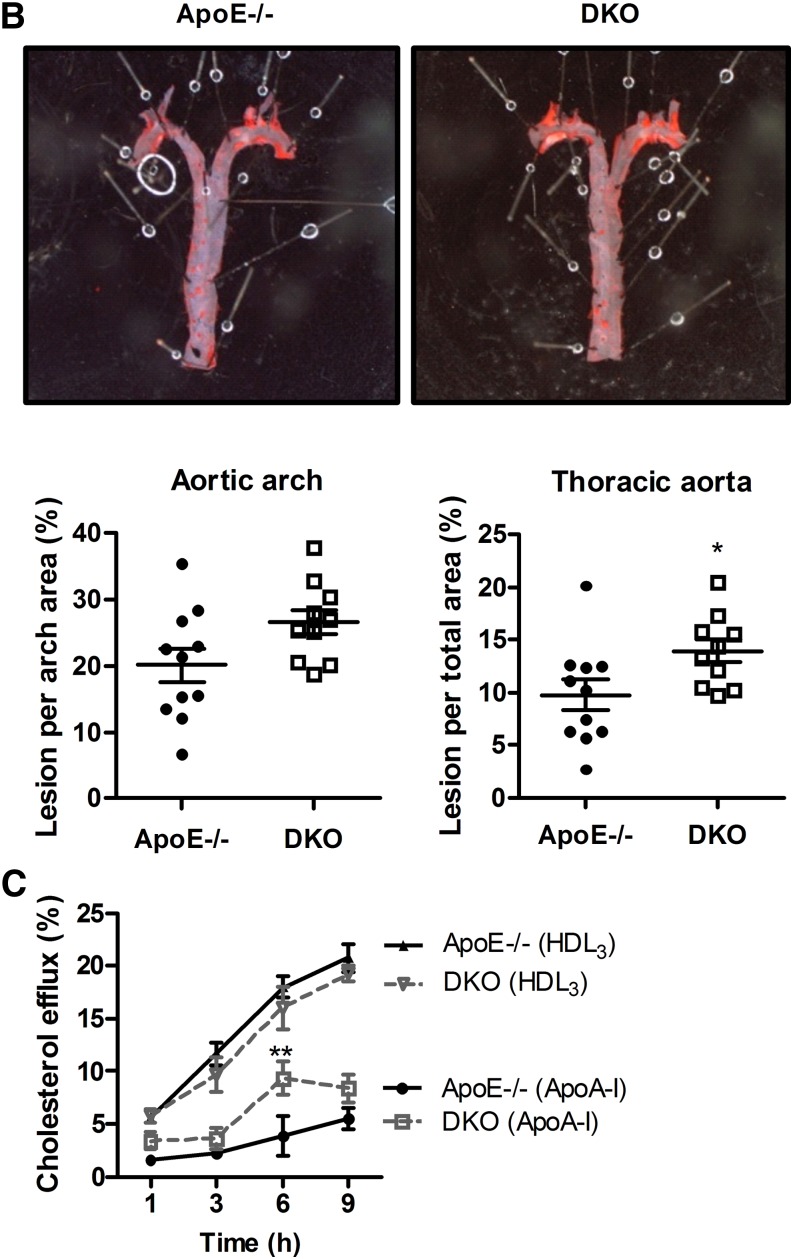
Unchanged atherosclerosis susceptibility by CGI-58 deficiency. ApoE^−/−^ and macCGI-58/ApoE-DKO mice were challenged with a HF/HCD for 10 weeks. A: Representative images of aortic valve sections stained with Oil Red O, Moma-2, and Masson’s trichrome for the detection of lipids, macrophages, and collagen, respectively. Magnification, ×40. Data represent mean values of 10 aortic valve sections per mouse. Bars represent the mean values of 9–11 mice per group. B: Representative images and quantification of Oil Red O-stained en face aorta. Data represent mean values ± SEM (n = 10–11). *P < 0.05. C: Cholesterol efflux to the extracellular acceptors ApoA-I and HDL_3_ expressed as the percentage of [^3^H]cholesterol transferred from macrophages to the medium. Data show the mean values ± SEM (n = 7).

To examine the consequences of CGI-58^−/−^ deficiency and the concomitant TG accumulation in myeloid cells on atherogenesis, we generated CGI-58^flox/flox^/ApoE^−/−^ (designated ApoE^−/−^) and macCGI-58/ApoE-DKO mice and challenged them with a HF/HCD to induce lesion formation. We observed no differences in body weight (not shown), plasma lipid parameters (**Table 1**), and plasma IL-6 concentrations (supplementary Fig. IVA) between DKO and ApoE^−/−^ mice. Macrophages from both genotypes show the same polarization (supplementary Fig. IVB), which is in contrast to what we observed in CGI-58^−/−^ compared with Wt macrophages. Visual inspection of Oil Red O-stained aortic root sections revealed no differences in lesion formation (**Fig. 6A**). Moma-2 and Masson’s trichrome staining, which were used to identify lesion macrophages and collagen, were comparable in sections from ApoE^−/−^ and DKO mice (Fig. 6A). Quantitative analysis of plaque development in aortic arches (en face analysis) revealed a 1.3-fold increase in lesion size in thoracic aortic arches of macCGI-58/ApoE-DKO mice (Fig. 6B).

**TABLE 1.** Plasma lipid parameters of overnight fasted ApoE^−/−^ and macCGI-58/ApoE-DKO mice fed chow diet or challenged with HF/HCD for 10 weeks

	Chow Diet		HF/HCD	
	ApoE^−/−^	DKO	ApoE^−/−^	DKO
TC (mg/dl)	247 ± 35.0	271 ± 37.6	1059 ± 379	1053 ± 261
FC (mg/dl)	69.9 ± 11.4	75.9 ± 12.5	324 ± 98.9	335 ± 74.5
TG (mg/dl)	108 ± 29.7	117 ± 27.3	73.7 ± 36.0	79.2 ± 37.3
FA (mmol/l)	1.3 ± 0.3	1.5 ± 0.3	0.9 ± 0.1	0.8 ± 0.2

Data represent mean values ± SEM (n = 10–11).

To investigate the effect of CGI-58 deficiency on cholesterol transport from macrophages to exogenous lipid acceptors, we measured cholesterol efflux to ApoA-I and HDL. As shown in Fig. 6C, cholesterol efflux from DKO macrophages to ApoA-I was increased after 6 h compared with ApoE^−/−^ macrophages, but unchanged to both acceptors at all other time points. This result is in line with unaltered mRNA levels of genes involved in cholesterol uptake (Cd36, SrB1) and efflux (Abca1, Abcg1) in CGI-58^−/−^ compared with Wt macrophages (not shown).

**Fig. 7.**
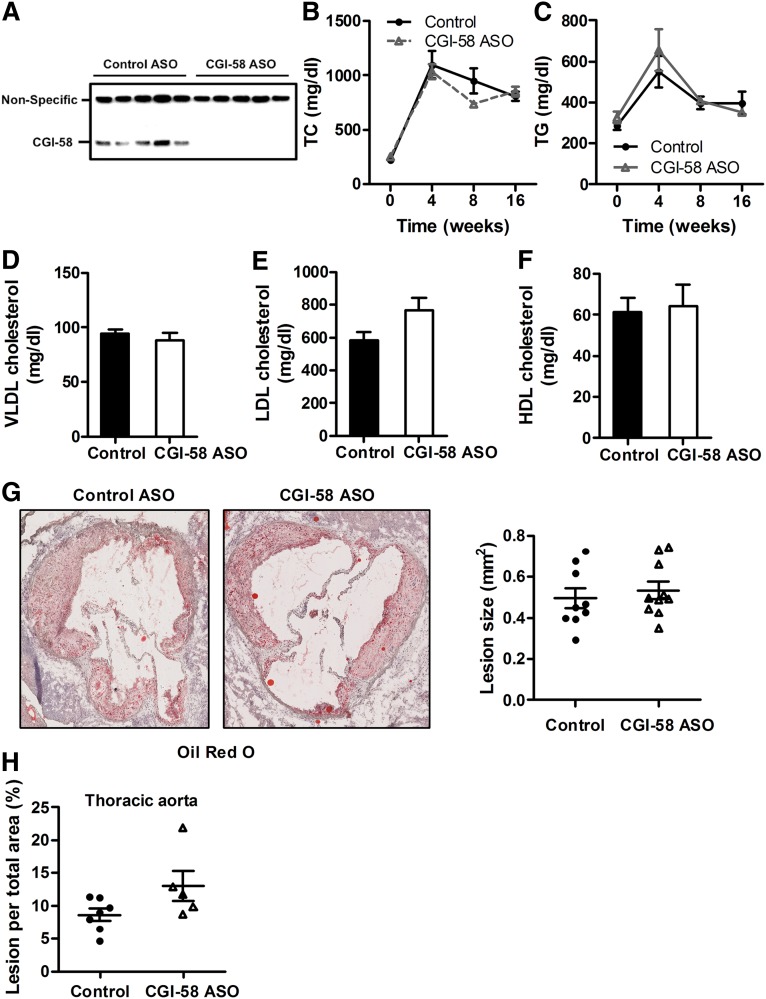
ASO-mediated knockdown of CGI-58 does not alter atherosclerosis susceptibility. A: ASO treatment effectively reduces macrophage expression of CGI-58. C57BL/6 mice maintained on a chow diet in conjunction with weekly injections (50 mg/kg) of either a nontargeting control ASO or CGI-58 for 6 weeks. Thereafter, thioglycolate-elicited peritoneal macrophages were isolated from control and CGI-58 ASO-treated mice (n = 5 per group), plated for 2 h, and Western blotting was conducted to examine CGI-58 protein expression in isolated macrophages. B–H: For atherosclerosis studies, 6-week-old male LDLR^−/−^ mice were fed a diet enriched in 0.2% (w/w) cholesterol and 20% of energy as lard for 16 weeks in conjunction with biweekly injections (25 mg/kg) of either a nontargeting control ASO or CGI-58 ASO. Total plasma cholesterol (TC) (B) and TG (C) concentrations during atherosclerosis progression. VLDL (D), LDL (E), and HDL (F) cholesterol levels following 16 weeks of diet induction. G: Representative images of Oil Red O-stained aortic valve sections (magnification, ×40) and quantification of cross-sectional aortic valve lesion area. H: En face morphometric analysis of total aortic lesion area. Data represent the mean ± SEM from four to ten mice per group.

To further interrogate the role of CGI-58 in atherosclerosis progression, we utilized ASO-mediated knockdown of CGI-58 in hyperlipidemic LDLR^−/−^ mice. ASO-mediated knockdown has previously been shown to reduce expression levels of CGI-58 in the liver, white adipose tissue, and kidney (9). Here we show that thioglycolate-elicited macrophages from ASO-treated mice have a marked reduction in macrophage CGI-58 expression as well (**Fig. 7A**). Using this system, we found that CGI-58 knockdown in macrophages and other organs has no significant effect on plasma TC or TG concentrations in LDLR^−/−^ mice (Fig. 7B, C). Likewise, CGI-58 knockdown did not alter circulating VLDL, LDL, or HDL cholesterol levels (Fig. 7D–F). Comparable to macCGI-58/ApoE-DKO mice (Fig. 6A), ASO-mediated knockdown of CGI-58 did not alter atherosclerosis burden in the aortic sinus (Fig. 7G) or thoracic aorta of LDLR^−/−^ mice as measured by en face morphometry (Fig. 7H). Collectively, these results indicate that diminished CGI-58 in macrophages, liver, and adipose tissue driven by CGI-58 ASO treatment or deficiency of CGI-58 in myeloid cells has minimal effects on atherosclerosis progression.

## DISCUSSION

Due to the functional changes in macrophages lacking ATGL (12–15), we proposed that the absence of its coactivator CGI-58 results in similar alterations of macrophage function. To investigate the consequences of CGI-58 deficiency in macrophages, we generated a mouse model lacking CGI-58 exclusively in myeloid cells including monocytes, mature macrophages, and granulocytes (macCGI-58^−/−^). These mice are viable with no apparent changes in skin phenotype. Compared with Wt mice, the lack of CGI-58 in myeloid cells does not affect body weight, lipid parameters, glucose levels, and glucose tolerance even when mice are challenged with WTD. Unchanged glucose tolerance in WTD-fed mice is contradictory to the results shown recently by Miao et al. (28), who reported impaired glucose tolerance in high-fat diet-fed male macCGI-58^−/−^ mice. This discrepancy is difficult to explain as both diets (WTD and high-fat diet) can be used to affect glucose tolerance. Because we have repeated the experiments in male mice that also lacked significant differences between the phenotypes (data not shown), a sex difference leading to the contradictory results can be excluded.

CGI-58 deficiency leads to decreased TG hydrolysis activity and a TG-rich lipid droplet accumulation in macrophages, identical to ATGL^−/−^ macrophages (12, 15). These results suggest that ATGL activity can be increased by CGI-58 as activator protein (2) also in macrophages. Unchanged CD36 and LPL mRNA as well as LPL activity argue against differences in FA uptake between CGI-58^−/−^ and Wt macrophages. Because LPL is responsible for the extracellular hydrolysis of lipoprotein-associated TG and the subsequent uptake of FAs in underlying cells and tissues, these results indicate that FAs generated by the action of LPL are taken up similarly by CGI-58^−/−^ and Wt macrophages. Unchanged TC and FC concentrations were associated with comparable CE hydrolase activities between Wt and CGI-58^−/−^ macrophages, as observed in ATGL^−/−^ macrophages (15). In contrast to our results, Miao et al. (28) found increased TC and FC concentrations and reduced CD36 mRNA expression in macrophages of male macCGI-58^−/−^ mice. Whether the differences in the two studies are due to sex-specific differences or cultivation conditions of the macrophages (LPDS versus FCS) remain to be elucidated.

Measurement of FA composition within TG of CGI-58^−/−^ macrophages revealed increased concentrations of all saturated, unsaturated, and polyunsaturated FAs analyzed with the highest change in arachidonic acid, oleic acid, and linoleic acid, respectively. FA composition has not been determined in ATGL^−/−^ macrophages, but in white adipose tissue of ATGL^−/−^ mice. Our findings are slightly different to these results (29), where the authors showed that ATGL hydrolyzes long-chain FA esters in vivo with a modest substrate preference for C16:1.

Fragmented mitochondria in ATGL^−/−^ macrophages are indicative of the mitochondrial apoptosis pathway being triggered as a consequence of defective lipolysis (12). We expected the same phenotype in CGI-58^−/−^ macrophages. Typical markers of programmed cell death, such as externalization of phosphatidylserine on the plasma membrane measured by flow cytometry after annexin V/PI costaining, mRNA and protein expression of pro-apoptotic markers, and lack of fragmented mitochondria prior to cell death, however, revealed that mitochondrial apoptosis is not induced in CGI-58^−/−^ macrophages. Absent CHOP protein expression and unaltered mRNA expression of the ER-resident chaperones Erdj4 and Pdi indicate that CGI-58 deficiency in macrophages does not cause ER stress. These results are different from findings in ATGL^−/−^ macrophages, where we observed induction of apoptosis (12) and ER stress (13). Interestingly, incubation of Wt macrophages with VLDL resulted in the same apoptotic phenotype and fragmentation of mitochondria as observed in ATGL^−/−^ macrophages (12). From these results, we had initially concluded that intracellular TG accumulation is linked to mitochondrial dysfunction and programmed cell death in macrophages. The results from the present work are therefore counterintuitive. Why TG accumulation leads to mitochondrial dysfunction in ATGL^−/−^ and VLDL-loaded Wt macrophages (12), but fails to affect CGI-58^−/−^ macrophages, is elusive. Because ATGL is present in CGI-58^−/−^ macrophages, it might be claimed that there is still basal ATGL-mediated TG hydrolase activity, which is sufficient to rescue the cell from mitochondrial apoptosis. However, macrophage TG concentrations are comparable between ATGL^−/−^ and CGI-58^−/−^ macrophages, suggesting other factors to be responsible for programmed cell death, mitochondrial dysfunction, and ER stress in ATGL^−/−^ macrophages.

The in vitro phagocytic capacity was affected by the lack of ATGL (15) and CGI-58 in a similar manner with reduced phagocytosis in glucose-containing and glucose-free medium compared with Wt macrophages. It has been demonstrated that hydrolysis of cellular TG by ATGL is necessary to produce FAs as ligands for PPAR activation and that ATGL deficiency leads to severely disrupted mitochondrial substrate oxidation and respiration (30). Although mRNA levels of PPARα target genes in macrophages were unchanged, decreased mitochondrial respiration might contribute to the reduced phagocytic capacity of CGI-58^−/−^ macrophages. Because the surface area of mitochondria was unaffected by CGI-58 deficiency, the reduced respiration is likely due to the decrease in FAs as energy substrate. A decreased OCR was also described in CGI-58-silenced RAW264.7 macrophages (28). Our in vitro finding of reduced phagocytosis could not be confirmed in vivo in macCGI-58^−/−^ mice, where we observed a trend to reduced phagocytosis, which, however, lacked statistical significance. We hypothesize that the reason for this discrepancy is the difference in the mouse models: in macCGI-58^−/−^ mice, CGI-58 is absent in myeloid cells including macrophages, in which phagocytosis is affected in vitro. In contrast, the decreased in vivo phagocytosis ability was demonstrated in whole-body ATGL^−/−^ mice (15). Phagocytosis is a highly energy demanding process; reduced FA concentrations in ATGL^−/−^ mice (7) versus unaltered FA levels in macCGI-58^−/−^ mice might explain the observed in vivo changes in phagocytic capacity.

Due to the pronounced differences observed between ATGL^−/−^ and CGI-58^−/−^ macrophages, we were particularly interested in the impact of macCGI-58 deficiency on atherosclerosis susceptibility. Unexpectedly, plaque formation, number of macrophages, and collagen content was identical in aortic root sections of macCGI-58/ApoE-DKO and ApoE^−/−^ mice. In line with these results, en face analyses of the aortic arch area revealed comparable lesion sizes in both genotypes. When we analyzed lesion per total area of the thoracic aorta, we found slightly increased plaque formation in DKO mice. In agreement, ASO-mediated knockdown of CGI-58 in LDLR^−/−^ mice did not alter atherosclerosis burden in the aortic root, but very modestly increased atherosclerosis in the aorta. Collectively, these findings are again contrary to what we observed in the absence of ATGL, where transfer of ATGL^−/−^ bone marrow into LDLR^−/−^ mice resulted in markedly reduced plaque formation (16). Although different mouse models were used in these two studies, our results indicate that the absence of ATGL or CGI-58 in myeloid cells results in different phenotypes: reduced lesion development by ATGL deficiency and unaltered or even slightly increased plaque formation by lack of CGI-58. This difference in plaque formation is unlikely due to differences in cholesterol efflux capacities of the cells, because macrophages lacking either ATGL or CGI-58 show comparable cholesterol efflux as control cells. The M2-like polarization of ATGL^−/−^ macrophages and the M1-like phenotype of CGI-58^−/−^ might contribute (at least in part) to the differences in plaque formation. In accordance with our results, Miao et al. (28) have recently shown that macrophage CGI-58 deficiency activates the ROS-NLRP3 inflammatory pathway to promote insulin resistance in mice. We found increased IL-6 secretion from LPS-treated CGI-58^−/−^ macrophages, suggesting that the lack of CGI-58 in macrophages affects the inflammatory response to LPS. This finding is in line with results from ASO-mediated CGI-58 knockdown experiments, in which lipid second messengers generated by CGI-58 were shown to be critically involved in maintaining the balance between inflammation and insulin action, thereby predicting a role of CGI-58 in cytokine signaling (31). On an ApoE^−/−^ background, however, plasma IL-6 concentrations and macrophage polarization markers were no longer different between mice that express or lack CGI-58 in macrophages. Because ApoE promotes macrophage conversion from M1 to the M2 phenotype (32), it might be speculated that the absence of ApoE directs macrophages into an M1-like phenotype, which cannot be further boosted by CGI-58 deficiency. In contrast, reduced plaque formation in aortic roots of LDLR^−/−^ mice transplanted with ATGL^−/−^ bone marrow was associated with decreased macrophage IL-6 concentrations (16).

The data presented in this study provide evidence that the TG-rich lipid droplet accumulation within ATGL^−/−^ macrophages is likely not the reason for attenuated atherosclerosis development as initially hypothesized. Although loss of CGI-58 in macrophages results in comparable TG accumulation, several results were different between CGI-58^−/−^ and ATGL^−/−^ macrophages: Absence of ER stress, mitochondrial apoptosis, and mitochondrial dysfunction, as well as M1-like polarization of CGI-58^−/−^ macrophages, and differences in atherosclerosis susceptibility argue for different and/or additional function(s) of CGI-58 in macrophages beside activation of ATGL. We conclude that lack of the enzyme (ATGL) induces a more severe phenotype than loss of the activator (CGI-58).

## Supplementary Material

Supplemental Data

## References

[1] McLaren J. E. Michael D. R. Ashlin T. G. Ramji D. P. 2011 Cytokines, macrophage lipid metabolism and foam cells: implications for cardiovascular disease therapy. Prog. Lipid Res. 50: 331–347. 21601592 10.1016/j.plipres.2011.04.002

[2] Lass A. Zimmermann R. Haemmerle G. Riederer M. Schoiswohl G. Schweiger M. Kienesberger P. Strauss J. G. Gorkiewicz G. Zechner R. 2006 Adipose triglyceride lipase-mediated lipolysis of cellular fat stores is activated by CGI-58 and defective in Chanarin-Dorfman syndrome. Cell Metab. 3: 309–319. 16679289 10.1016/j.cmet.2006.03.005

[3] Zechner R. Zimmermann R. Eichmann T. O. Kohlwein S. D. Haemmerle G. Lass A. Madeo F. 2012 FAT SIGNALS–lipases and lipolysis in lipid metabolism and signaling. Cell Metab. 15: 279–291. 22405066 10.1016/j.cmet.2011.12.018 PMC3314979

[4] Schweiger M. Lass A. Zimmermann R. Eichmann T. O. Zechner R. 2009 Neutral lipid storage disease: genetic disorders caused by mutations in adipose triglyceride lipase/PNPLA2 or CGI-58/ABHD5. Am. J. Physiol. Endocrinol. Metab. 297: E289–E296. 19401457 10.1152/ajpendo.00099.2009

[5] Radner F. P. Streith I. E. Schoiswohl G. Schweiger M. Kumari M. Eichmann T. O. Rechberger G. Koefeler H. C. Eder S. Schauer S. 2010 Growth retardation, impaired triacylglycerol catabolism, hepatic steatosis, and lethal skin barrier defect in mice lacking comparative gene identification-58 (CGI-58). J. Biol. Chem. 285: 7300–7311. 20023287 10.1074/jbc.M109.081877 PMC2844178

[6] Fischer J. Lefevre C. Morava E. Mussini J. M. Laforet P. Negre-Salvayre A. Lathrop M. Salvayre R. 2007 The gene encoding adipose triglyceride lipase (PNPLA2) is mutated in neutral lipid storage disease with myopathy. Nat. Genet. 39: 28–30. 17187067 10.1038/ng1951

[7] Haemmerle G. Lass A. Zimmermann R. Gorkiewicz G. Meyer C. Rozman J. Heldmaier G. Maier R. Theussl C. Eder S. 2006 Defective lipolysis and altered energy metabolism in mice lacking adipose triglyceride lipase. Science. 312: 734–737. 16675698 10.1126/science.1123965

[8] Lefèvre C. Jobard F. Caux F. Bouadjar B. Karaduman A. Heilig R. Lakhdar H. Wollenberg A. Verret J. L. Weissenbach J. 2001 Mutations in CGI-58, the gene encoding a new protein of the esterase/lipase/thioesterase subfamily, in Chanarin-Dorfman syndrome. Am. J. Hum. Genet. 69: 1002–1012. 11590543 10.1086/324121 PMC1274347

[9] Brown J. M. Betters J. L. Lord C. Ma Y. Han X. Yang K. Alger H. M. Melchior J. Sawyer J. Shah R. 2010 CGI-58 knockdown in mice causes hepatic steatosis, but prevents diet-induced obesity and glucose intolerance. J. Lipid Res. 51: 3306–3315. 20802159 10.1194/jlr.M010256 PMC2952571

[10] Lord C. C. Brown J. M. 2012 Distinct roles for alpha-beta hydrolase domain 5 (ABHD5/CGI-58) and adipose triglyceride lipase (ATGL/PNPLA2) in lipid metabolism and signaling. Adipocyte. 1: 123–131. 23145367 10.4161/adip.20035 PMC3492958

[11] Zierler K. A. Zechner R. Haemmerle G. 2014 Comparative gene identification-58/alpha/beta hydrolase domain 5: more than just an adipose triglyceride lipase activator? Curr. Opin. Lipidol. 25: 102–109. 24565921 10.1097/MOL.0000000000000058 PMC4170181

[12] Aflaki E. Radovic B. Chandak P. G. Kolb D. Eisenberg T. Ring J. Fertschai I. Uellen A. Wolinski H. Kohlwein S. D. 2011 Triacylglycerol accumulation activates the mitochondrial apoptosis pathway in macrophages. J. Biol. Chem. 286: 7418–7428. 21196579 10.1074/jbc.M110.175703 PMC3044998

[13] Aflaki E. Doddapattar P. Radovic B. Povoden S. Kolb D. Vujic N. Wegscheider M. Koefeler H. Hornemann T. Graier W. F. 2012 C16 ceramide is crucial for triacylglycerol-induced apoptosis in macrophages. Cell Death Dis. 3: e280. 22419109 10.1038/cddis.2012.17 PMC3317349

[14] Aflaki E. Balenga N. A. Luschnig-Schratl P. Wolinski H. Povoden S. Chandak P. G. Bogner-Strauss J. G. Eder S. Konya V. Kohlwein S. D. 2011 Impaired Rho GTPase activation abrogates cell polarization and migration in macrophages with defective lipolysis. Cell. Mol. Life Sci. 68: 3933–3947. 21533980 10.1007/s00018-011-0688-4 PMC3214256

[15] Chandak P. G. Radovic B. Aflaki E. Kolb D. Buchebner M. Frohlich E. Magnes C. Sinner F. Haemmerle G. Zechner R. 2010 Efficient phagocytosis requires triacylglycerol hydrolysis by adipose triglyceride lipase. J. Biol. Chem. 285: 20192–20201. 20424161 10.1074/jbc.M110.107854 PMC2888432

[16] Lammers B. Chandak P. G. Aflaki E. Van Puijvelde G. H. Radovic B. Hildebrand R. B. Meurs I. Out R. Kuiper J. Van Berkel T. J. 2011 Macrophage adipose triglyceride lipase deficiency attenuates atherosclerotic lesion development in low-density lipoprotein receptor knockout mice. Arterioscler. Thromb. Vasc. Biol. 31: 67–73. 21030715 10.1161/ATVBAHA.110.215814 PMC3063945

[17] Zierler K. A. Jaeger D. Pollak N. M. Eder S. Rechberger G. N. Radner F. P. Woelkart G. Kolb D. Schmidt A. Kumari M. 2013 Functional cardiac lipolysis in mice critically depends on comparative gene identification-58. J. Biol. Chem. 288: 9892–9904. 23413028 10.1074/jbc.M112.420620 PMC3617289

[18] Clausen B. E. Burkhardt C. Reith W. Renkawitz R. Forster I. 1999 Conditional gene targeting in macrophages and granulocytes using LysMcre mice. Transgenic Res. 8: 265–277. 10621974 10.1023/a:1008942828960

[19] Brown J. M. Chung S. Sawyer J. K. Degirolamo C. Alger H. M. Nguyen T. Zhu X. Duong M. N. Wibley A. L. Shah R. 2008 Inhibition of stearoyl-coenzyme A desaturase 1 dissociates insulin resistance and obesity from atherosclerosis. Circulation. 118: 1467–1475. 18794388 10.1161/CIRCULATIONAHA.108.793182 PMC2716169

[20] Sattler W. Puhl H. Hayn M. Kostner G. M. Esterbauer H. 1991 Determination of fatty acids in the main lipoprotein classes by capillary gas chromatography: BF3/methanol transesterification of lyophilized samples instead of Folch extraction gives higher yields. Anal. Biochem. 198: 184–190. 1838668 10.1016/0003-2697(91)90526-y

[21] Schweiger M. Eichmann T. O. Taschler U. Zimmermann R. Zechner R. Lass A. 2014 Measurement of lipolysis. Methods Enzymol. 538: 171–193. 24529439 10.1016/B978-0-12-800280-3.00010-4 PMC4018506

[22] Pfaffl M. W. Horgan G. W. Dempfle L. 2002 Relative expression software tool (REST) for group-wise comparison and statistical analysis of relative expression results in real-time PCR. Nucleic Acids Res. 30: e36. 11972351 10.1093/nar/30.9.e36 PMC113859

[23] Kratzer A. Buchebner M. Pfeifer T. Becker T. M. Uray G. Miyazaki M. Miyazaki-Anzai S. Ebner B. Chandak P. G. Kadam R. S. 2009 Synthetic LXR agonist attenuates plaque formation in apoE−/− mice without inducing liver steatosis and hypertriglyceridemia. J. Lipid Res. 50: 312–326. 18812595 10.1194/jlr.M800376-JLR200 PMC2636920

[24] Tischner D. Manzl C. Soratroi C. Villunger A. Krumschnabel G. 2012 Necrosis-like death can engage multiple pro-apoptotic Bcl-2 protein family members. Apoptosis. 17: 1197–1209. 22971741 10.1007/s10495-012-0756-8 PMC4918797

[25] Marciniak S. J. Yun C. Y. Oyadomari S. Novoa I. Zhang Y. Jungreis R. Nagata K. Harding H. P. Ron D. 2004 CHOP induces death by promoting protein synthesis and oxidation in the stressed endoplasmic reticulum. Genes Dev. 18: 3066–3077. 15601821 10.1101/gad.1250704 PMC535917

[26] Martinez F. O. Sica A. Mantovani A. Locati M. 2008 Macrophage activation and polarization. Front. Biosci. 13: 453–461. 17981560 10.2741/2692

[27] Sharda D. R. Yu S. Ray M. Squadrito M. L. De Palma M. Wynn T. A. Morris S. M. Jr Hankey P. A. 2011 Regulation of macrophage arginase expression and tumor growth by the Ron receptor tyrosine kinase. J. Immunol. 187: 2181–2192. 21810604 10.4049/jimmunol.1003460 PMC4042865

[28] Miao H. Ou J. Ma Y. Guo F. Yang Z. Wiggins M. Liu C. Song W. Han X. Wang M. 2014 Macrophage CGI-58 deficiency activates ROS-inflammasome pathway to promote insulin resistance in mice. Cell Reports. 7: 223–235. 24703845 10.1016/j.celrep.2014.02.047 PMC4040312

[29] Eichmann T. O. Kumari M. Haas J. T. Farese R. V. Jr Zimmermann R. Lass A. Zechner R. 2012 Studies on the substrate and stereo/regioselectivity of adipose triglyceride lipase, hormone-sensitive lipase, and diacylglycerol-O-acyltransferases. J. Biol. Chem. 287: 41446–41457. 23066022 10.1074/jbc.M112.400416 PMC3510842

[30] Haemmerle G. Moustafa T. Woelkart G. Büttner S. Schmidt A. van de Weijer T. Hesselink M. Jaeger D. Kienesberger P. C. Zierler K. 2011 ATGL-mediated fat catabolism regulates cardiac mitochondrial function via PPAR-alpha and PGC-1. Nat. Med. 17: 1076–1085. 21857651 10.1038/nm.2439 PMC3244833

[31] Lord C. C. Betters J. L. Ivanova P. T. Milne S. B. Myers D. S. Madenspacher J. Thomas G. Chung S. Liu M. Davis M. A. 2012 CGI-58/ABHD5-derived signaling lipids regulate systemic inflammation and insulin action. Diabetes. 61: 355–363. 22228714 10.2337/db11-0994 PMC3266405

[32] Baitsch D. Bock H. H. Engel T. Telgmann R. Muller-Tidow C. Varga G. Bot M. Herz J. Robenek H. von Eckardstein A. 2011 Apolipoprotein E induces antiinflammatory phenotype in macrophages. Arterioscler. Thromb. Vasc. Biol. 31: 1160–1168. 21350196 10.1161/ATVBAHA.111.222745 PMC3529398

