# Supplementary material for: Deletion of CGI-58 or adipose triglyceride lipase differently affects macrophage function and atherosclerosis
Source: J Lipid Res. 2014 Dec;55(12):2562–75. doi: 10.1194/jlr.M052613 (PMC4242449; doi:10.1194/jlr.M052613)
Supplement: Supplemental Data [file supp_55_12_2562__index.html]

Deletion of CGI-58 or adipose triglyceride lipase differently affects macrophage function and atherosclerosis — Deletion of CGI-58 or adipose triglyceride lipase differently affects macrophage function and atherosclerosis — Supplemental Data 

# Deletion of CGI-58 or adipose triglyceride lipase differently affects macrophage function and atherosclerosis

## Supplemental Data

**Files in this Data Supplement:**

- Supplement - supplemental methods supplemental figures S1-S4
