# Supplementary material for: Deletion of CGI-58 or adipose triglyceride lipase differently affects macrophage function and atherosclerosis
Source: J Lipid Res. 2014 Dec;55(12):2562–75. doi: 10.1194/jlr.M052613 (PMC4242449; doi:10.1194/jlr.M052613)
Supplement: Supplemental Data [file supp_M052613_jlr.M052613-1.pdf]

## Supplemental information

**Deletion of CGI-58 or adipose triglyceride lipase differently affects macrophage function and atherogenesis (Goeritzer et al.)**

**Primer sequences for real time PCR**

Primer sequences were used as follows:

HPRT-fwd: 5′-GCCTGTATCCAACACTTCG-3′

HPRT-rev: 5′-GCTGACCTGCTGGATTACA-3′

CGI-58-fwd: 5′-GGTTAAGTCTAGTGCAGC-3′

CGI-58-rev: 5′-AAGCTGTCTCACCACTTG-3′

Bcl-XL-fwd: 5′-GACAAGGAGATGCAGGTATTGG-3′

Bcl-XL-rev: 5′-TCCCGTAGAGATCCACAAAAGT-3′

Mcl-1-fwd: 5′-AAAGGCGGCTGCATAAGTC-3′

Mcl-1-rev: 5′-CTGGCGGTATAGGTCGTCCTC-3′

Pdi-fwd: 5′- CAAGATCAAGCCCCACCTGAT -3′

Pdi-rev: 5′-AGTTCGCCCCAACCAGTACTT-3′

Erdj4-fwd: 5′-CCCCAGTGTCAAACTGTACCAG-3′

Erdj4-rev: 5′-AGCGTTTCCAATTTTCCATAAATT-3′

Cpt1α-fwd: 5′-CTCCGCCTGAGCCATGAAG-3′

Cpt1α -rev: 5′-CACCAGTGATGATGCCATTCT-3′

Aox-fwd: 5′-AGATTGGTAGAAATTGCTGCAAAA-3′

Aox-rev: 5′-ACGCCACTTCCTTGCTCTTC-3′

Vlcad-fwd: 5′-CTACTGTGCTTCAGGGACAAC-3′

Vlcad-rev: 5′-CAAAGGACTTCGATTCTGCCC-3′

Mcad-fwd: 5′-GCAACTGCCCGCAAGTTT-3′

Mcad-rev: 5′-TACTCCCCGCTTTTGTCATATTC-3′

Gro-1-fwd: 5′-CTGGGATTCACCTCAAGAACATC-3′

Gro-1-rev: 5′-CAGGGTCAAGGCAAGCCTC-3′

Mcp-1-fwd: 5′-ACTGAAGCCAGCTCTCTCTTCCTC-3′

Mcp-1-rev: 5′-TTCCTTCTTGGGGTCAGCACAGAC-3′

Mcp-2-fwd: 5′-TCTACGCAGTGCTTCTTTGCC-3′

Mcp-2-rev: 5′-AAGGGGGATCTTCAGCTTTAGTA-3′

Ccl5-fwd: 5′-GCTGCTTTGCCTACCTCTCC-3′

Ccl5-rev: 5′-TCGAGTGACAAACACGACTGC-3′

Mrc-1-fwd: 5′-GCTGAATCCCAGAAATTCCGC-3′

Mrc-1-rev: 5′-ATCACAGGCATACAGGGTGAC-3′

ATGL-fwd: 5′-GCCACTCACATCTACGGAGC-3′

ATGL-rev: 5′-GACAGCCACGGATGGTCTTC-3′

HSL-fwd: 5′-GCTGGTGACACTCGCAGAAG-3′

HSL-rev: 5′-TGGCTGGTGTCTCTGTGTCC-3′

LPL-fwd: 5′-ACATTCCCGTTACCGTCCATC-3′

LPL-rev: 5′-GGACCCCTGAAGACACAG-3′

LAL-fwd: 5′-CGGCTTGCTGGCAGATTCTA-3′

LAL-rev: 5′-GTGCAGCCTTGAGAATGACC-3′

**Confocal analysis of mitochondrial area**

To label mitochondria, macrophages were seeded on glass coverslips in 6-well plates. After 24 h, cells were incubated with MitoTracker® Red probes (25 nM) in a loading buffer containing 135 mM NaCl, 5 mM KCl, 2 mM $CaCl_2$, 1 mM $MgCl_2$, 10 mM Hepes, 2.6 mM $NaHCO_3$, 440 µM $KH_2PO_4$, 340 µM $Na_2HPO_4$, 10 mM D-glucose, 0.1% vitamins, 0.2% essential amino acids, and 1% penicillin/streptomycin (pH 7.4). Staining solution was replaced after 20 min with fresh loading buffer. Z-scans of stained cells were performed on a Nipkow-disk-based array confocal laser-scanning microscope, equipped with VoxCell Scan® (VisiTech, Visitron Systems, Puchheim, Germany) and controlled by VisiView Premier Acquisition software (Visitron Systems). Stained cells were imaged with a 100X objective (α-Plan-Fluar 100x/1.45 NA Oil, Zeiss Microsystems, Oberkochen, Germany) using the 514 nm

diode laser (50 mW, Visitron Systems) for illumination. Emitted light was filtered at 568 nm (Chroma Technology Corp., Bellows Falls, VT, USA) and images were taken with a CCD camera (CoolSNAP-HQ, Roper Scientific Corp., Martinsried, Germany). Mitochondrial area analysis was performed with MetaMorph 7.7.0.0 software (Visitron Systems).

**Western blotting of proteins involved in oxidative phosphorylation**

Protein samples of lysed macrophages from the different genotypes (40 µg protein/lane) were separated by SDS-PAGE (15%). Proteins were transferred to polyvinylidene difluoride or nitrocellulose membranes. Blots were incubated with a monoclonal anti-mouse antibody against cyclooxygenase 4 (1:500) (Santa Cruz, Heidelberg, Germany), a monoclonal anti-mouse antibody cocktail (MitoProfile® Membrane Integrity WB Antibody Cocktail; 1:200; Abcam Cambridge, UK), and with a polyclonal anti-rabbit antibody against GAPDH (1:1000) (Cell Signaling Technology, Danvers, MA). HRP-conjugated goat anti-rabbit (1:5000) or rabbit anti-mouse antibodies (1:1000) (Dako, Glostrup, Denmark) were visualized by enhanced chemiluminescence detection (Clarity™ Western ECL Substrate; Bio-Rad, Vienna, Austria) using a ChemiDoc™ MP Imaging System (Bio-Rad, Vienna, Austria).

**Supplemental Figures**

**Figure S1**

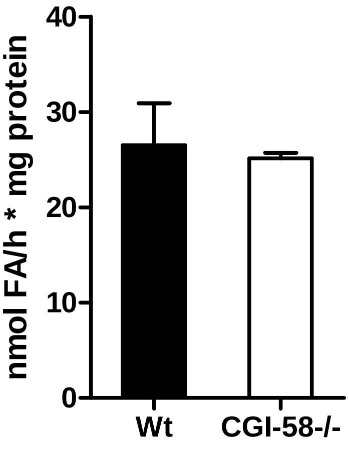


**Figure S1: Unchanged neutral cholesteryl ester hydrolase activity in CGI-58-/- macrophages.** CE hydrolase activities were assayed in lysates from Wt and CGI-58-/- macrophages. Data are presented as mean values (n=5) performed in duplicate + SEM.

**Figure S2**

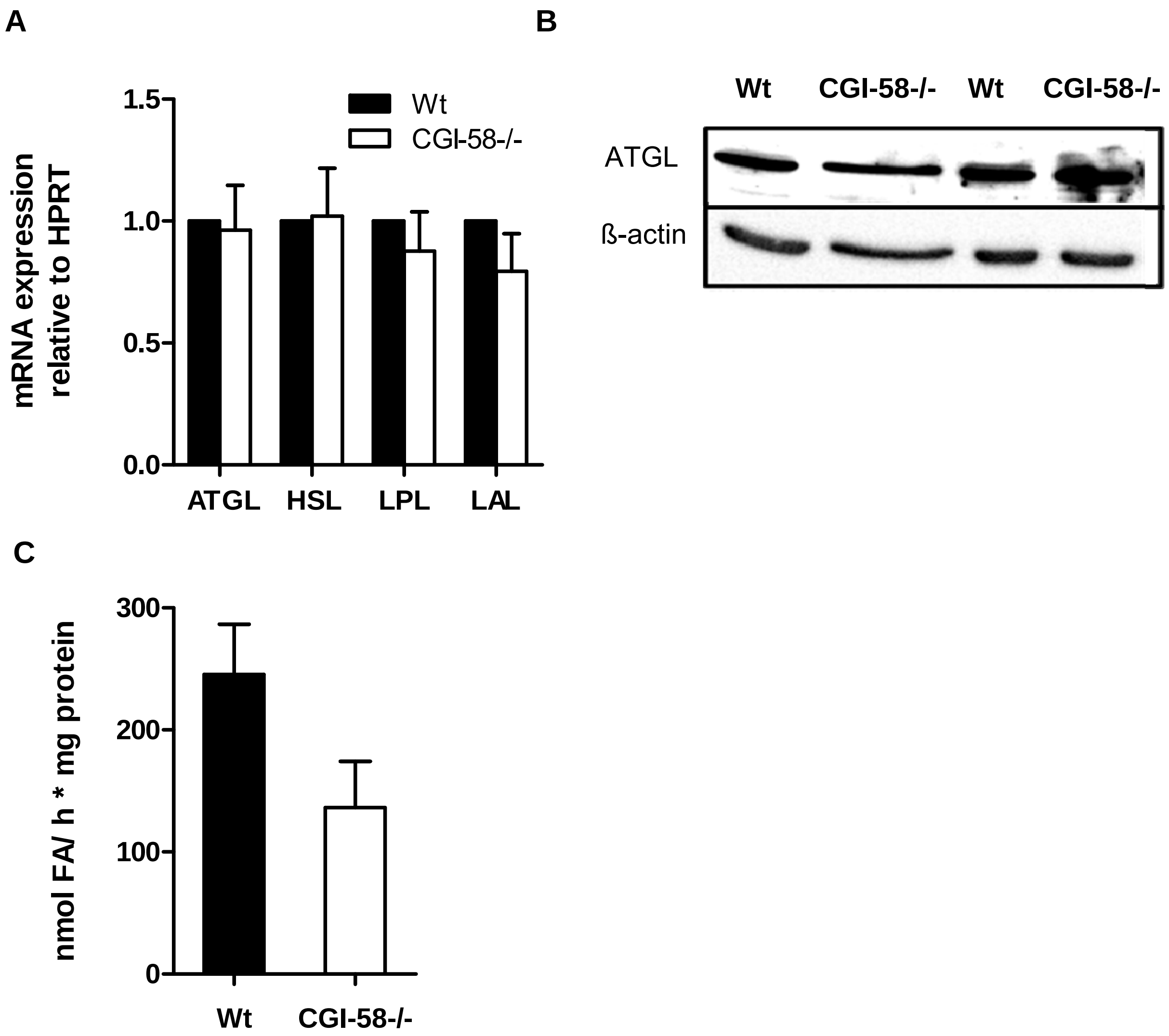


**Figure S2: Unchanged mRNA expression of genes involved in TG hydrolysis and comparable protein expression of ATGL in Wt and CGI-58-/- macrophages.** (A) mRNA expression of ATGL, HSL, LPL, and LAL in Wt and CGI-58-/- macrophages was determined by real time PCR, including normalization to HPRT. mRNA expression in Wt macrophages was arbitrarily set to 1. Data are expressed as mean values (n=5) performed in duplicate + SEM. (B) Cell lysates of macrophages (50 µg of protein per lane) were separated by SDS-PAGE. Protein expression of ATGL was determined by Western blotting. (C) Wt and CGI-58-/- macrophages were incubated with [$^3$H]triolein for 1 h and assayed for LPL activity. Data are shown as mean values (n=4-6) performed in duplicate + SEM.

**Figure S3**

A

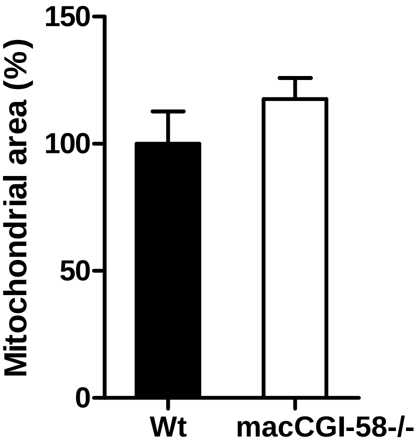


B

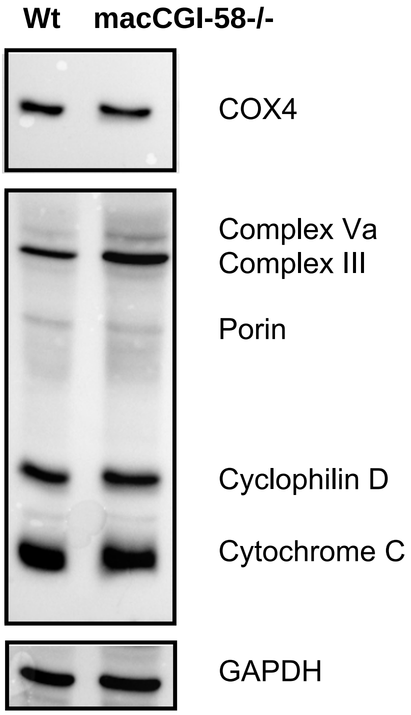


**Figure S3: Unchanged mitochondrial area and expression of proteins involved in oxidative phosphorylation in CGI-58-/- macrophages.** (A) Mitochondrial area in MitoTracker® Red-stained CGI-58-/- macrophages is expressed as percentage of mitochondrial area relative to Wt cells. Data are shown as means (n=8) + SEM. (B) Cell lysates of macrophages (40 µg of protein per lane) were separated by SDS-PAGE. Western blotting analysis of cyclooxygenase 4 (COX4), Complex Va (ATP5A), and Complex III Core I (ubiquinol-cytochrome C reductase). Antibodies against porin, cyclophilin D, and cytochrome C were present in the antibody mixture (MitoProfile) and expression of these proteins are shown as control. Expression of GAPDH was determined as loading control.

**Figure S4**

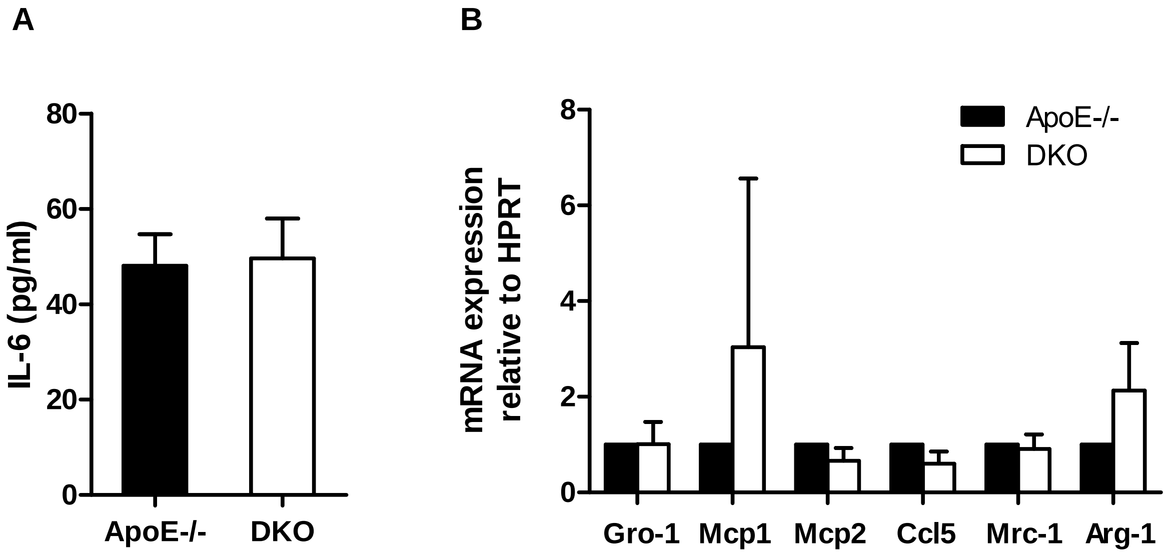


**Figure S4: Comparable plasma IL-6 and macrophage polarization in macCGI-58/ApoE-DKO and ApoE-/- mice.** ApoE-/- and macCGI-58/ApoE-DKO mice were challenged with a HF/HCD for 10 weeks. (A) Plasma IL-6 levels were determined by ELISA. Data represent mean values (n=8) + SEM. (B) mRNA expression of Gro-1, Mcp-1, Mcp-2, Ccl5, Mrc-1, and Arg-1 in ApoE-/- and macCGI-58/ApoE-DKO macrophages was determined by real time PCR, including normalization to HPRT. Expression in Wt macrophages was arbitrarily set to 1. Data are presented as mean values (n=4-5) performed in duplicate + SEM.
